# Supplementary material for: Sphagnum increases soil’s sequestration capacity of mineral-associated organic carbon via activating metal oxides
Source: Nat Commun. 2023 Aug 19;14:5052. doi: 10.1038/s41467-023-40863-0 (PMC10439956; doi:10.1038/s41467-023-40863-0)
Supplement: Supplementary file 1 — Supplementary Information [file 41467_2023_40863_MOESM1_ESM.pdf]

Supplementary Information for

Sphagnum increases soil's sequestration capacity of mineral-associated organic carbon via activating metal oxides

Yunpeng Zhao[1,2,3], Chengzhu Liu[1,2,3], Xingqi Li[1,2,3], Lixiao Ma[1,2,3], Guoqing Zhai[1,2,3], Xiaojuan Feng[1,2,3*]

[1]*State Key Laboratory of Vegetation and Environmental Change, Institute of Botany, Chinese Academy of Sciences, Beijing 100093, China*

[2]*China National Botanical Garden, Beijing 100093, China*

[3]*College of Resources and Environment, University of Chinese Academy of Sciences, Beijing 100049, China*

Correspondence to: X. Feng (email: xfeng@ibcas.ac.cn)

This PDF file includes:

Figures S1 to S8.

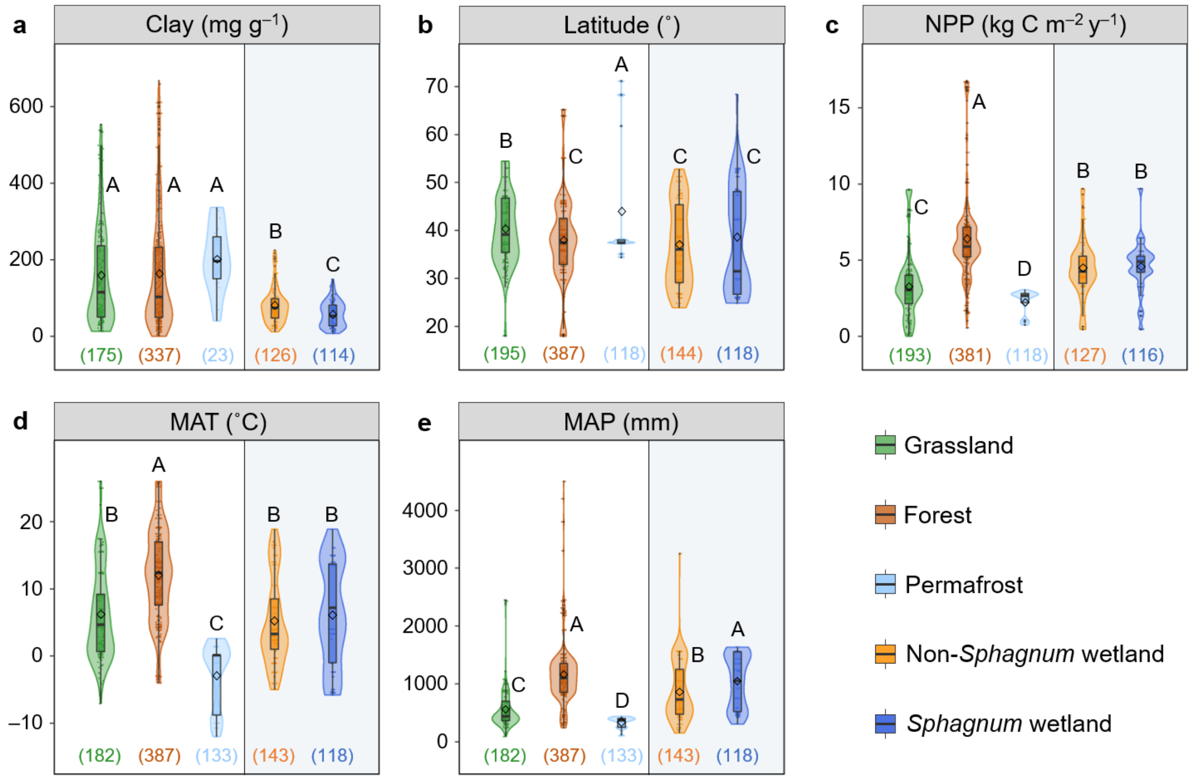

Fig. S1. Comparison of environmental variables of the studied sites across various terrestrial ecosystems. (a) Clay; **(b)** latitude; **(c)** NPP; **(d)** MAT; **(e)** MAP. NPP, net primary production; MAT, mean annual temperature; MAP, mean annual precipitation. Numbers in parenthesis indicate the number of samples. The violin plot shows the distribution of data. The solid line and rhombus in the box mark the median and mean of each dataset, respectively. The upper and lower ends of boxes denote the 0.25 and 0.75 percentiles, respectively. The upper and lower whisker caps denote the 1.5 interquartile range of upper and lower quartile, respectively. Dots indicate the value of samples. Dots outside whiskers indicate outliers. Upper-case letters indicate different levels among various ecosystems ($p < 0.05$;

one-way ANOVA). Raw data are given in Supplementary Data1.

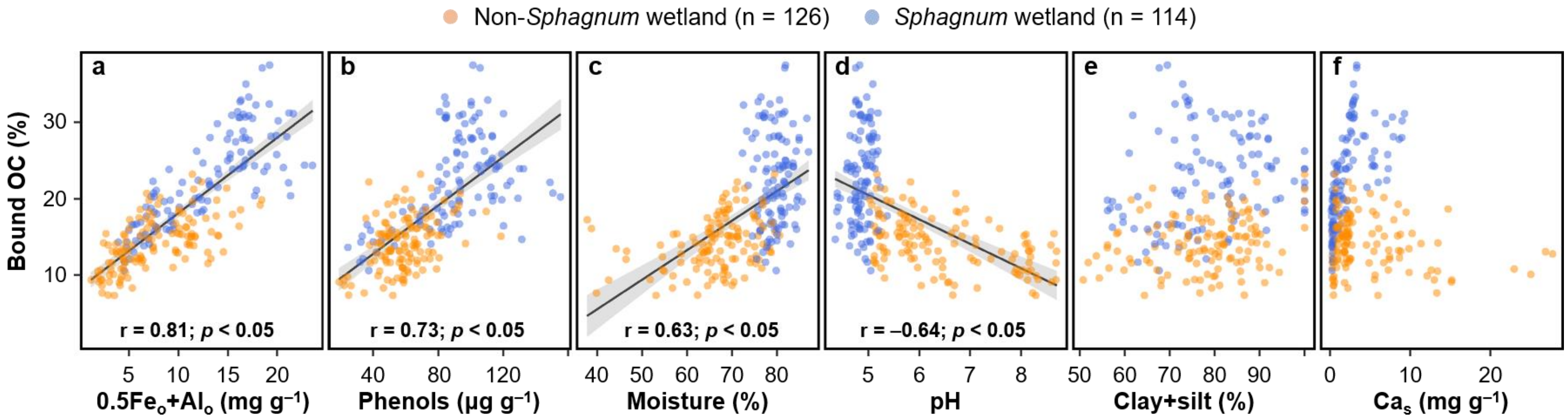

Fig. S2. Spearman's correlations of bound OC with **(a)** 0.5Fe_o+Al_o; **(b)** phenols; **(c)** moisture; **(d)** pH; **(e)** clay+silt; **(f)** Ca_s. Bound OC, organic carbon bound to reactive metal oxides extracted by the citrate-bicarbonate-dithionite method; Fe_o and Al_o, oxalate-extractable iron and aluminum; 0.5Fe_o+Al_o, weight-normalized contents of Fe_o and Al_o; Ca_s, sulfate-extractable Ca. Black solid lines indicate linear regressions (n = 240; $p < 0.05$). The shaded areas represent the 95% confidence intervals.

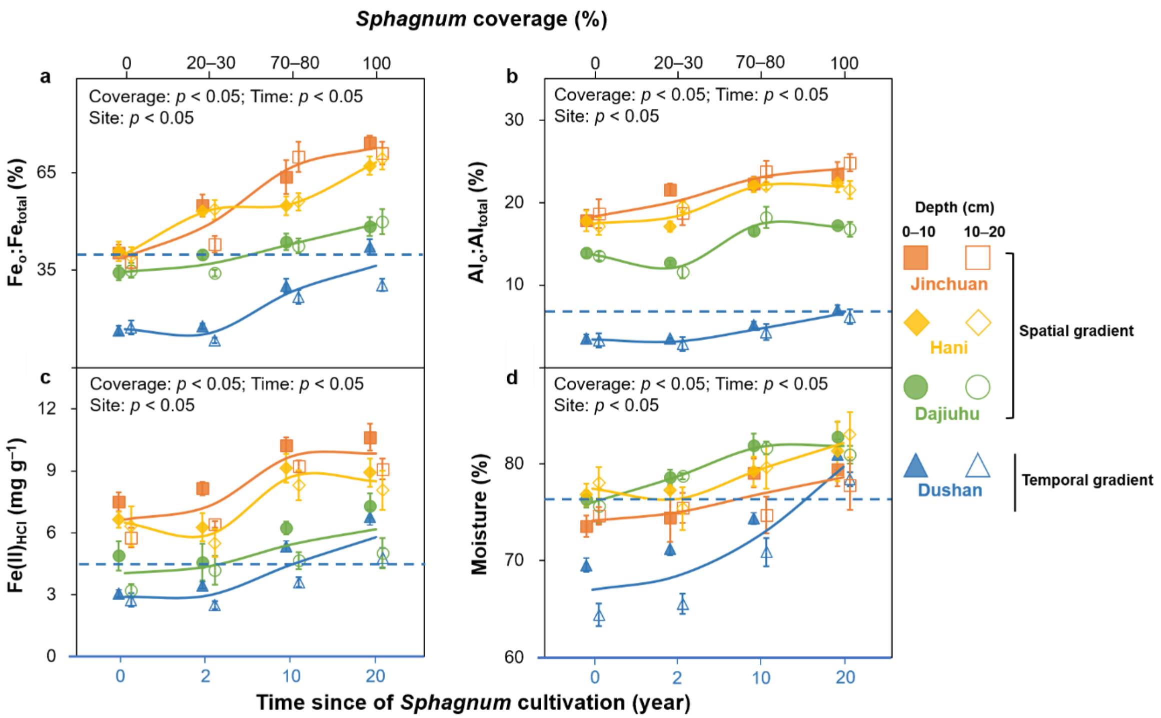

Fig. S3. Dynamic increases of (a) Fe_o:Fe_{total}, (b) Al_o:Al_{total}, (c) $Fe(II)_{HCl}$ and (d) moisture contents with *Sphagnum* expansion. Fe_o and Al_o, oxalate-extractable iron and aluminum; Fe_{total} and Al_{total}, soil total iron and aluminum; $Fe(II)_{HCl}$, soil ferrous iron extracted by 0.5-M hydrochloric acid (HCl). X-axis represents *Sphagnum* coverage (%) at the top (Jinchuan, Hani and Dajiuhu) and the time since of *Sphagnum* cultivation at the bottom (Dushan). Blue dashed line indicates the average value of pristine *Sphagnum*-dominated wetland in Dushan. Mean values are shown with standard error (n = 3). Only significant effects are noted ($p < 0.05$; one-way ANOVA).

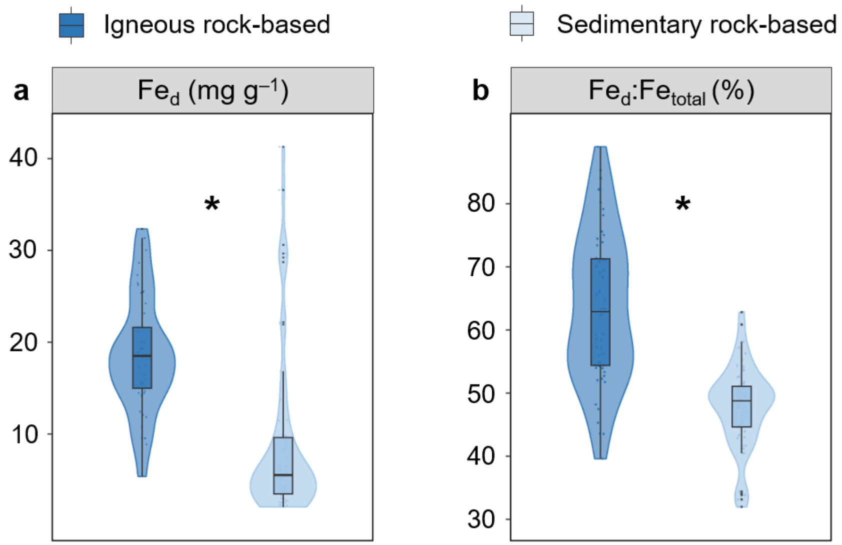

Fig. S4. Transformable Fe (hydr)oxides in igneous rock- and sedimentary rock-based wetlands. (a) Fe_d; **(b)** Fe_d:Fe_{total}. Fe_d, dithionite-extractable iron; Fe_{total}, soil total iron; The violin plot shows the distribution of data. The solid line in the box marks the median of each dataset. The upper and lower ends of boxes denote the 0.25 and 0.75 percentiles, respectively. The upper and lower whisker caps denote the 1.5 interquartile range of upper and lower quartile, respectively. Dots indicate the value of samples. Dots outside whiskers indicate outliers. Black asterisk denotes significant difference between igneous rock-based (n = 56) and sedimentary rock-based (n = 58) wetlands ($p < 0.05$; one-way ANOVA).

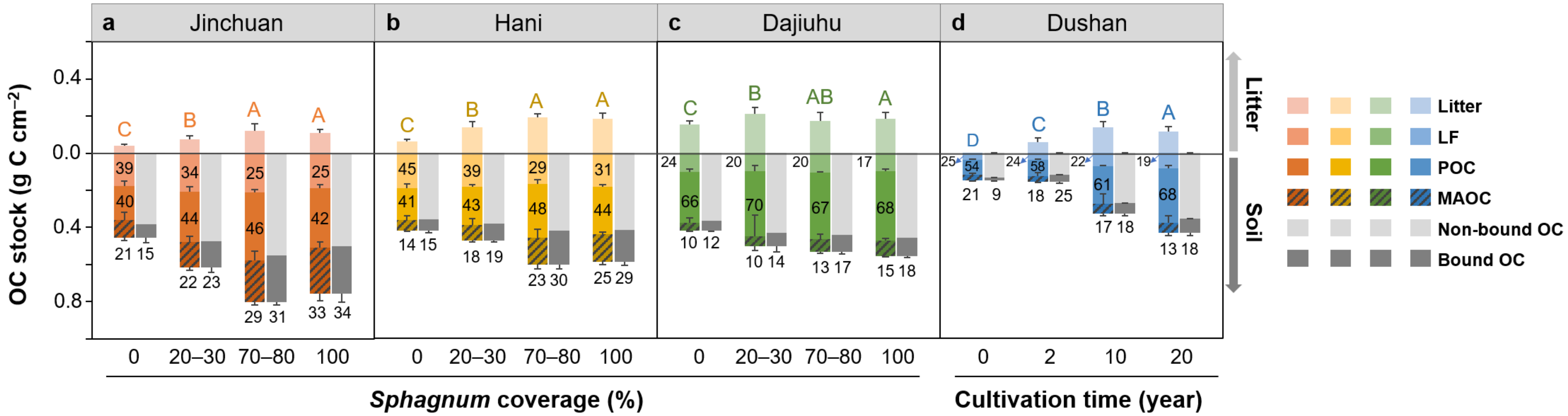

Fig. S5. Change in organic carbon (OC) stock in the litter layer, LF, POC, MAOC and bound OC in (a) Jinchuan, (b) Hani, (c) Dajiuhu and (d) Dushan with *Sphagnum* expansion. LF, light fraction (density < 1.6 g cm^{-3}); POC, particulate organic carbon (density > 1.6 g cm^{-3} and size > 53 μm); MAOC, mineral-associated organic carbon (density > 1.6 g cm^{-3} and size < 53 μm); bound OC, organic carbon bound to reactive metal oxides extracted by the citrate-bicarbonate-dithionite method. Numbers represent the percentage of OC stock for each fraction relative to total SOC stocks. Mean values are shown with standard error (n = 3). Upper-case letters indicate different levels among different coverages or cultivation time ($p < 0.05$; one-way ANOVA).

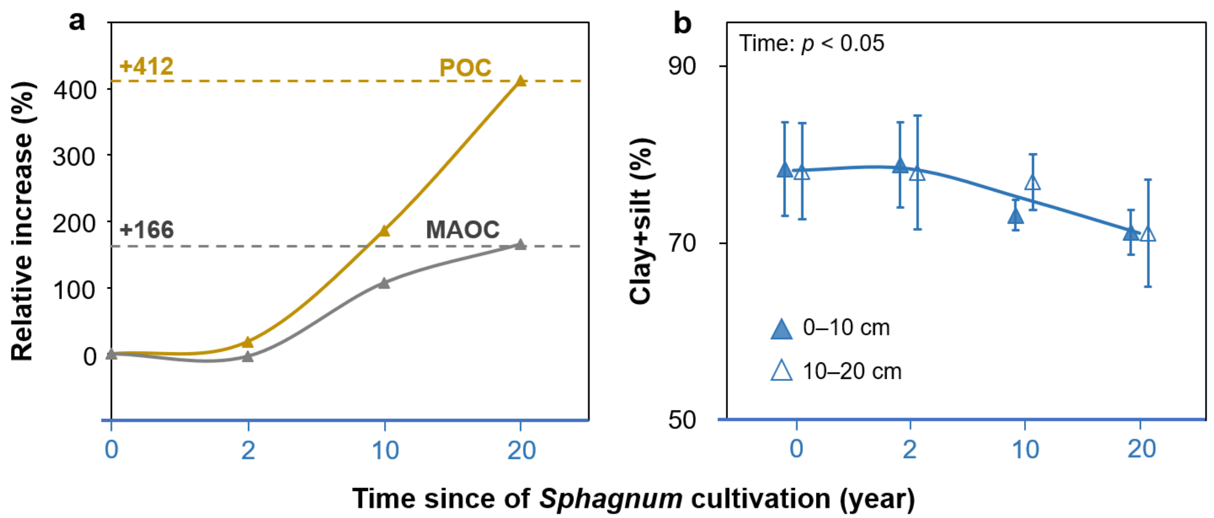

Fig. S6. (a) Relative increases of POC and MAOC with *Sphagnum* cultivation; **(b)** change in soil clay+silt content with *Sphagnum* cultivation. POC, particulate organic carbon (density > 1.6 g cm^{-3} and size > 53 μm); MAOC, mineral-associated organic carbon (density > 1.6 g cm^{-3} and size < 53 μm). Mean values are shown with standard error (n = 3). Only significant effects are noted ($p < 0.05$; one-way ANOVA).

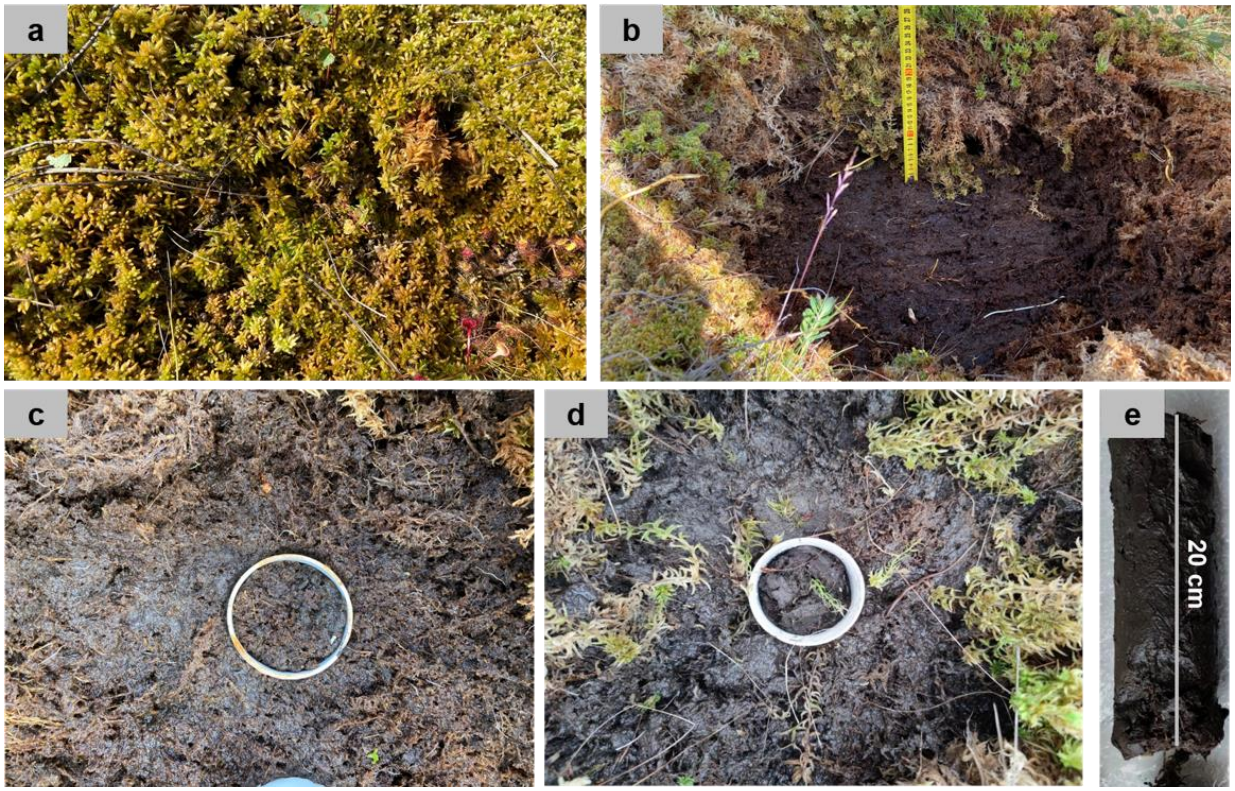

Fig. S7. Photos of our surveyed *Sphagnum* wetlands (in Dajiuhu and Dushan). (a) *Sphagnum* moss; **(b)** the moss layer; **(c)**, **(d)** soils under *Sphagnum* moss; **(e)** soil column collected by PVC pipes.

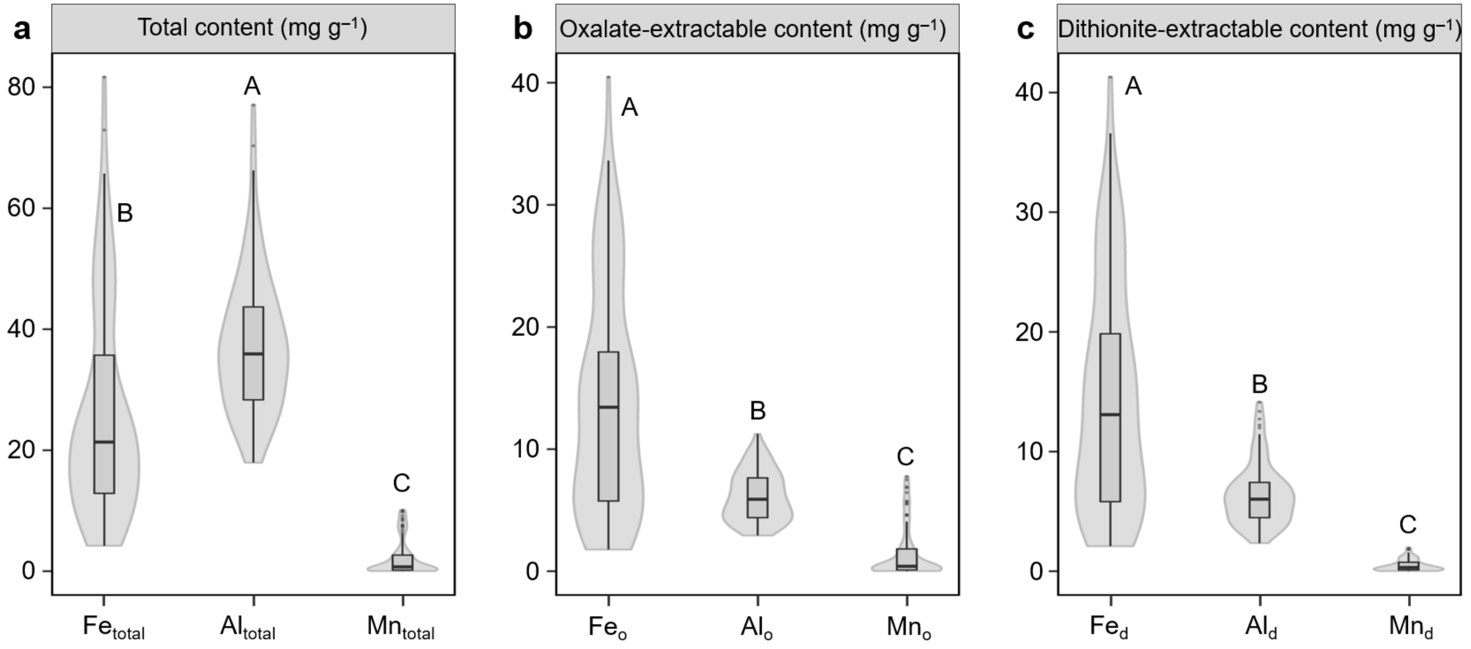

Fig. S8. Soil (a) total, (b) oxalate-extractable and (c) dithionite-extractable Fe, Al and manganese (Mn) contents in our surveyed *Sphagnum* wetlands (n = 114). Fe_{total}, Al_{total} and Mn_{total}, soil total iron, aluminum and manganese; Fe_o, Al_o and Mn_o, oxalate-extractable iron, aluminum and manganese; Fe_d, Al_d and Mn_d, dithionite-extractable iron, aluminum and manganese. The violin plot shows the distribution of data. The solid line in the box marks the median of each dataset. The upper and lower ends of boxes denote the 0.25 and 0.75 percentiles, respectively. The upper and lower whisker caps denote the 1.5 interquartile range of upper and lower quartile, respectively. Dots indicate the value of samples. Dots outside whiskers indicate outliers. Upper-case letters indicate different levels among Fe, Al and Mn contents ($p < 0.05$; one-way ANOVA).
