# Supplementary material for: Sphagnum increases soil’s sequestration capacity of mineral-associated organic carbon via activating metal oxides
Source: Nat Commun. 2023 Aug 19;14:5052. doi: 10.1038/s41467-023-40863-0 (PMC10439956; doi:10.1038/s41467-023-40863-0)
Supplement: Supplementary file 3 — Description of Additional Supplementary Files [file 41467_2023_40863_MOESM3_ESM.pdf]

Description of Additional Supplementary Files:

Supplementary Data 1: Information on sampling sites and raw data for Meta-analysis.

Supplementary Data 2: Soil properties of our surveyed wetlands.
